# Dissemination of an Evidence-based Program to Reduce Fear of Falling, South Carolina, 2006-2009

**DOI:** 10.5888/pcd9.110093

**Published:** 2012-05-24

**Authors:** Gerhild Ullmann, Harriet G. Williams, Cora F. Plass

**Affiliations:** Author Affiliations: Gerhild Ullmann, Clemson University, Clemson, South Carolina; Cora F. Plass, South Carolina Department of Health and Environmental Control, Columbia, South Carolina.

## Abstract

**Introduction:**

Falls among older adults are a serious public health issue, and fear of falling can limit mobility, which in turn increases fall risk. A Matter of Balance/Volunteer Lay Leader Model is an evidence-based program designed to address fear of falling. The objective of this study was to describe implementation, dissemination, and outcomes of this program in 3 regions of South Carolina with a predominantly African American and largely underserved population.

**Methods:**

We developed partnerships throughout the state, organized master and lay leader trainings, and documented numbers of lay leaders, programs offered, demographic characteristics of participants, program fidelity, and attendance. Outcome measures were self-reported confidence to prevent and manage falls and a quantitative measure of functional mobility. Both measures were assessed at baseline and after program completion.

**Results:**

Older adults (N = 235) attended 18 classes at 16 sites. Barriers to implementation were program teams' limited familiarity with the concept of evidence-based programs and the importance of adhering to program content. Facilitators were state-level leadership and a history of state, regional, and local groups collaborating successfully on other projects. Outcomes indicated greater confidence in managing falls and carrying out activities of daily living. Mobility improved significantly, suggesting a reduced risk for falls.

**Conclusion:**

Evidence-based programs such as A Matter of Balance/Volunteer Lay Leader Model can be successfully disseminated in underserved areas. Outcomes indicate that participation in fall prevention programs can benefit groups of predominantly African American older adults.

## MEDSCAPE CME

Medscape, LLC is pleased to provide online continuing medical education (CME) for this journal article, allowing clinicians the opportunity to earn CME credit.

This activity has been planned and implemented in accordance with the Essential Areas and policies of the Accreditation Council for Continuing Medical Education through the joint sponsorship of Medscape, LLC and Preventing Chronic Disease. Medscape, LLC is accredited by the ACCME to provide continuing medical education for physicians. 

Medscape, LLC designates this Journal-based CME activity for a maximum of 1 **AMA PRA Category 1 Credit(s)™**. Physicians should claim only the credit commensurate with the extent of their participation in the activity.

All other clinicians completing this activity will be issued a certificate of participation. To participate in this journal CME activity: (1) review the learning objectives and author disclosures; (2) study the education content; (3) take the post-test with a 70% minimum passing score and complete the evaluation at www.medscape.org/journal/pcd (4) view/print certificate.

**Release date: May 23, 2012; Expiration date: May 23, 2013**

### Learning Objectives

Upon completion of this activity, participants will be able to:

- Describe implementation of AMOB/VLL, an evidence-based program designed to address fear of falling
- Describe barriers and facilitators to implementation of AMOB/VLL
- Describe outcomes of implementing AMOB/VLL

**CME EDITOR**

Nancy Saltmarsh, Editor, *Preventing Chronic Disease*. Disclosure: Nancy Saltmarsh has disclosed no relevant financial relationships.

**CME AUTHOR**

Laurie Barclay, MD. Freelance writer and reviewer, Medscape, LLC. Disclosure: Laurie Barclay, MD, has disclosed no relevant financial relationships. 

**AUTHORS AND CREDENTIALS**

Disclosures: Gerhild Ullmann, PhD; Harriet G. Williams, PhD; Cora F. Plass, MSW have disclosed no relevant financial relationships.

Affiliations: Gerhild Ullmann, Clemson University, Clemson, South Carolina; Harriet G. Williams, University of South Carolina, Department of Exercise Science, Blatt Center, Columbia, South Carolina; Cora F. Plass, South Carolina Department of Health and Environmental Control, Columbia, South Carolina.

## Introduction

Falls and fall-related injuries are a public health issue; they often have serious consequences for older adults and can lead to loss of independence. Every year, 36% to 40% of adults aged 65 or older fall at least once (1,2); falls are the leading cause of unintentional injuries and injury deaths among adults aged 65 or older. The total direct cost of fall-related nonfatal injuries for this population in 2000 in the United States was approximately $19 billion (3). The prevalence of falls and fall-related injuries in South Carolina is similar to that for the United States (1).

Falls and fall-related injuries are not necessarily a result of aging. Environmental, physical, and behavioral factors contribute to increased fall risk. Fear of falling is an additional factor contributing to falls (4,5); it can affect gait and is often a barrier to participation in physical activity (6,7). Inactivity further increases the fear of and risk for falls, creating a downward spiral that can lead to loss of function and disability. Fall prevention programs are effective in reducing fall risk factors and thereby the rate and number of falls (2,7-9). Providing community-based, affordable, and accessible fall-prevention programs to older adults of all ages and functional abilities is a proactive approach to prevent falls.

As part of an Administration on Aging grant to mobilize states to implement evidence-based programs in local communities, we partnered with the Aging Network (Area Agencies on Aging and aging service providers), the Lieutenant Governor's Office on Aging (LGOA), the South Carolina Department of Health and Environmental Control (DHEC), and the University of South Carolina (USC) School of Public Health to implement a 3-year project in South Carolina (2006-2009). In year 2, we implemented A Matter of Balance/Volunteer Lay Leader (AMOB/VLL) Model (10,11), an evidence-based program designed to address fear of falling in older adults (10,12-14). The primary objective of this study was to describe the implementation and dissemination of AMOB/VLL in a predominantly African American and largely underserved population in 3 regions where agencies and stakeholders had limited experience with such programs. The secondary objective was to examine potential benefits of the program using quantitative measures of mobility and perceived ability to manage falls.

## Methods

The study was a quasi-experimental design with pre-post assessments and no control group. The institutional review boards of DHEC and USC approved study procedures and protocols. All participants could withdraw from the study at any time and signed a written consent before programs and testing began.

### Program implementation

We disseminated AMOB/VLL in 3 regions of South Carolina: Appalachia, Pee Dee, and Trident (Lowcountry). The Appalachia region includes the urban areas Greenville, Spartanburg, and Anderson and surrounding rural areas in 6 counties. The Trident region includes Berkeley, Charleston, and Dorchester counties. Metropolitan Charleston is surrounded by rural, underserved, and geographically isolated areas. The Pee Dee region in northeastern South Carolina encompasses 6 counties and is a rural underserved area with a large African American population. Program sites included churches, hospitals, older-adult centers, and Council on Aging nutrition centers.

AMOB/VLL uses a cognitive-behavioral approach that focuses on identifying and modifying fall risk factors (eg, home environment, exercise behaviors, poor falls management), teaches that falls are not an inevitable consequence of aging, and uses theory to overcome misconceptions and raise confidence of participants (10-12). The program provides strategies to cope with fear of falling and opportunities to learn practical ways to reduce fall risk. AMOB/VLL is designed for small groups (8-12 participants) and involves 8 two-hour, structured sessions (10,11).

We created a 3-tiered organizational structure consisting of an active, engaged, and interactive group of people and organizations. Tiers included a state-level team, a regional team, and local service providers to serve as implementation sites. The state team consisted of representatives from South Carolina DHEC (State Health Unit), LGOA (State Aging Unit), and the USC School of Public Health. Key responsibilities of the state team were consulting with regional project teams and providing technical assistance to these teams in developing partnerships, implementing programs, and evaluating program delivery. The regional teams (1 for each area studied) included representatives from regional health departments, Area Agencies on Aging, and local County Councils on Aging. The third tier included local aging service provider organizations (eg, local community centers, retirement communities, Council on Aging nutrition centers, and churches and other faith-based organizations).

Representatives from the state tier made up the core team, which provided overall leadership for the project. Major activities of the core team and regional teams are described in the box.

**Box. Major Activities of the Core Team and Regional Teams for A Matter of Balance/Volunteer Lay Leader (AMOB/VLL) Project, South Carolina, 2006-2009**

**Table:** 

**Core team**
Organize kick-off meeting to introduce the concept of evidence-based programs to different groups.
Provide information about program fidelity and its importance in implementing programs in the community.
Determine target areas in the state to offer initial programs.
Organize and schedule conference calls or meetings to discuss issues, share ideas, and resolve concerns about implementing and evaluating AMOB/VLL programs.
Help develop PowerPoint presentations, brochures, and other health promotion materials to create awareness of programs and to recruit participants.
Help develop tools for evaluating program outcomes.
Train program coordinators to conduct pre-post assessments.
Analyze outcomes from completed programs and share information with local service providers.
Coordinate a master training session with the Maine Health Partnership for Healthy Aging.
Monitor program activities to assist in assessing program fidelity.

**Regional teams**
Identify potential community partners to help implement AMOB/VLL programs.
Work to develop partnerships.
Help identify and recruit potential lay leaders to lead classes.
Schedule classes.
Assign lay leaders to instruct classes.

Training staff to implement and conduct AMOB/VLL programs involved 2 phases: preparing master trainers and training lay leaders to lead classes.

Regional teams identified candidates for master trainers. National trainers from the Maine Department of Health and Human Services, originator of AMOB/VLL, provided a master trainer session (2 days, 16 hours) in South Carolina at the outset of the project (10,11). Representatives from 2 regions attended; the third region already had master trainers. Each prospective master trainer received an AMOB/VLL manual with information about content and timelines for sessions, protocols for pre-post assessments, handouts for participants, and forms for documenting class activities. National trainers reviewed the content of each session. Trainees participated in a teach-back practice assignment to ensure mastery of program content. Following the training, master trainers conducted 2 initial AMOB/VLL classes; members of the core team monitored the classes and helped master trainers present thorough and appropriate program content. Master trainers then assumed the role of recruiting and training lay leaders to conduct AMOB/VLL classes.

Regional teams and service provider organizations identified potential lay leaders to implement local programs. Regional program coordinators and master trainers organized 2-day trainings (16 hours) for lay leaders, using the same format as for master trainings. Master trainers helped lay leaders throughout the implementation of programs in their areas.

We used several strategies to recruit participants: enrolling participants from sites that served older adults, placing advertisements in local newspapers, attending health fairs, distributing fliers and brochures throughout the local community, and speaking at Council on Aging sites and to local faith-based groups for older adults. After several programs were completed, word of mouth became another recruiting tool.

### Measures

We used first and last session surveys and the attendance form designed for the project by states involved in the broader Agency on Aging initiative (10,11). We also obtained information about annual income and health status at baseline.

To monitor implementation and dissemination of programs, teams met regularly to review and discuss ongoing activities and to document major barriers and facilitators through reports from site program coordinators and minutes of meetings. We tracked numbers of programs offered by region, lay leaders trained, total participants, and attendance. People who attended 5 or more of 8 AMOB/VLL sessions were considered to be completers and to gain benefits from the program (12). All programs used an introductory session to provide program information and conduct pretesting.

We monitored program fidelity by using checklists designed to evaluate the degree to which content of AMOB/VLL programs was carried out according to program protocol. Fidelity checklists were developed for each session, including the introductory session, and highlighted the main topics of session content, provided a space to check whether that part of the session had been completed, and allowed for comments about deviations from session content. Lay leaders used fidelity checklists as a guide in carrying out each session's activities and completed them immediately after each session. Lay leaders' objectivity in using fidelity checklists was examined during implementation of the AMOB/VLL program in Lee County, South Carolina. In this instance, independent observers visited sessions periodically, reviewed activities, and completed checklists. Comparison of independent observers' checklists with those of lay leaders indicated 98% agreement.

We measured outcomes with the Falls Management Scale (FMS) and Timed Up and Go (TUG), a quantitative assessment of mobility performance (reported intra- and inter-rater reliability were 0.98 and 0.99, respectively) (11,12,15). Both outcomes were assessed at baseline and in the last session. The 5-item FMS measures perception of the ability to manage fall risk and to handle falls if they occur (11,12). The scale includes items that ask how sure respondents are about reducing falls, increasing strength, or becoming steadier on their feet. Scores on the 4-point Likert scale range from 1 for "very sure" to 4 for "not at all sure" (11). Lower scores indicate higher perceived ability to manage falls and fall risk. The TUG requires participants to stand up from a seated position, walk 3 meters, return, and sit down (15). Average time for 2 trials is recorded. Faster times indicate better mobility. Taking more than 14 seconds to complete the TUG indicates some limitation of mobility and increased risk for falls, loss of independence, and institutionalization (16).

Not all sites provided complete data on all aspects of programs. We ascertained the number of attendees and degree of program completion by using attendance data. To assess barriers to program implementation, we reviewed reports from regional teams and notes from team meetings.

To evaluate fidelity to program content, we examined fidelity checklists from all programs using these guidelines and reviewed and categorized written comments from lay leaders about omissions or deviations. We calculated the percentage of adherence to program content on the basis of checked items and summarized comments.

### Statistical analysis

For analyses of intervention outcomes, we used only data from participants who completed both preassessments and postassessments and we applied mixed models for repeated measures to compare performances. All analyses were performed using SAS version 9.1 (SAS Institute, Inc, Cary, North Carolina). Significance was set at *P* < .05. All models controlled for age.

## Results

We received demographic information on 150 participants from 13 programs in 2 regions. Demographic data from 5 programs were missing. Not all 150 participants completed all survey questions. Fifty-seven percent of participants were African American, 62% had an annual income of less than $15,000, and 69% had a high school education or less (Table 1).

**Table 1:** Baseline Characteristics of 150 Participants in A Matter of Balance/Volunteer Lay Leader Program, South Carolina (Appalachian and Pee Dee Regions), 2006-2009

Characteristic^a^	Value^b^
**Age, mean (SD), y (n = 130)**	75.4 (9.7)
**Sex (n = 130)**	
Male	18 (14)
Female	112 (86)
**Race (n = 129)**	
African American	74 (57)
White	51 (40)
Other	4 (3)
**Marital status (n = 137)**	
Married	27 (20)
Widowed	77 (56)
Other^c^	33 (24)
**Education (n = 138)**	
High school graduate or less	95 (69)
Some college or college graduate	34 (25)
Some graduate school or graduate	9 (7)
**Annual household income, $ (n = 100)**	
<15,000	62 (62)
15,000-24,999	23 (23)
25,000-49,999	12 (12)
>50,000	3 (3)
**Chronic disease^d^ (n = 132)**	
Hypertension	93 (70)
Arthritis	72 (55)
Diabetes	38 (29)
**FMS score,^e^ mean (SD) (n = 108)**	2.3 (0.1)
**TUG,^f^ mean (SD), seconds (n = 113)**	13.0 (5.7)

Abbreviations: FMS, Falls Management Scale (11); SD, standard deviation; TUG, Timed Up and Go (15).

^a^ Values for n indicate number of participants who reported on this item or completed the task and thus do not total 150.

^b^ Values are n (%) except where otherwise indicated.

^c^ Single, divorced, or separated.

^d^ Multiple answers allowed.

^e^ The 5-item FMS measures perception of the ability to manage fall risk and to handle falls if they occur (11,12).

^f^ The TUG requires participants to stand up from a seated position, walk 3 meters, return, and sit down (15).

Fifteen lay leaders conducted 18 AMOB/VLL classes in 3 regions at 16 sites (Table 2). The 18 AMOB/VLL classes reached 235 older adults. Attendance data were available for 215 participants; 175 participants (81%) attended at least 5 of 8 sessions.

**Table 2:** Attendance at A Matter of Balance/Volunteer Lay Leader Program, by Region, South Carolina, 2006-2009

Region	No. of Programs	No. of Participants Who Attended ≤5 of 8 Sessions	No. of Participants Who Attended ≤1 Session
Appalachian	4	41	44
Lowcountry	3	31	52
Pee Dee	11	103^a^	139^a^
South Carolina	18	175	235

^a^ For 20 participants in 1 program, no attendance form was available; thus, we were unable to determine the number of people who started or attended at least 5 sessions of this program.

Major barriers included lack of awareness about the availability of programs, insufficient knowledge of the potential benefits of such programs, and lack of easy access to program sites. Barriers for program leaders were related to implementation of programs, such as lack of knowledge or skills regarding 1) evidence-based programs, 2) program fidelity and its importance, and 3) how to administer, collect, and record pre-post assessment data. Facilitating factors included the state-level leadership in guiding the project; the history of the state, regional, and local groups working successfully together on other projects; the structure of the Area Agencies on Aging; the recruitment of participants through these agencies; and the commitment of local groups to providing their facilities for use in delivering the program.

We received fidelity checklists for 13 programs; 117 sessions were conducted (13 programs A- 9 sessions) and 116 fidelity checklists were completed. More than 80% of program content was delivered appropriately in all 13 programs. Nine programs covered more than 90% of program content.

For the 108 FMS and 113 TUG participants for whom data were available, analyses showed significant improvement in self-reported falls management (FMS) and objectively measured mobility (TUG). Age-adjusted mean (standard deviation [SD]) scores on the FMS improved from 2.3 (0.07) at baseline to 1.8 (0.07) post-intervention (*P* < .001); TUG age-adjusted performance times decreased from a mean (SD) of 13.0 (0.47) seconds to 11.7 (0.47) seconds (*P* < .001).

## Discussion

AMOB/VLL programs can be successfully implemented and disseminated in rural and underserved areas where agencies have limited experience with evidence-based programs. The program was successfully disseminated to 3 regions. The popularity of programs in terms of numbers of programs offered and numbers of attendees suggests that older adults in South Carolina, many of whom are poor, have low literacy, and often are not aware of available community-based programs, are interested in and will participate in such programs when encouraged.

We reached a majority–African American population with lower education and income levels than participants in AMOB/VLL programs in other states (17). Dissemination efforts in Texas indicated that 30% of participants belonged to racial/ethnic minority groups, 40% had an annual income of $15,000 or less, and 82% had completed high school. In South Carolina, 57% of participants were African American, 62% had annual incomes less than $15,000, and 69% had a high school education or less.

Major barriers were helping regional teams understand the concept of evidence-based programs and the importance of adhering to program content. We used various approaches and devoted considerable time through training sessions to develop this understanding. The success of these efforts is reflected in the number of programs implemented and the number of lay leaders who volunteered for training and instructed classes.

Evaluating program fidelity and outcomes was necessary; therefore, we attempted to ensure that lay leaders were comfortable with procedures for administering pre-post assessments and recording and transferring data (many lay leaders had not previously used a stopwatch). No demographic data were available from 5 programs; in addition, attendance forms were missing for 20 participants. Still, we had data on most participants who completed 13 programs. Deviation from delivery of prescribed program content was minimal and occurred most frequently in the final 2 sessions. This was due, in part, to the participation of guest health professionals in these sessions. AMOB/VLL content encourages the use of guest speakers, but often their participation took time away from other program content.

Facilitating factors included the leadership provided by the state agencies in guiding area partners in planning and implementing programs and the structure of the Area Agencies on Aging for delivering services to older adults. These groups had a history of collaboration. Another factor was the decision to recruit participants from organizations that already served older adults. Commitment of partner groups to offer use of facilities as sites for program implementation was integral to project success. Such commitment facilitated participant recruitment and expedited program implementation.

To our knowledge, this is the first time that AMOB/VLL program outcomes were evaluated by using a quantitative assessment of mobility. Although exercise was only 1 component of the AMOB/VLL program, we found a significant improvement in mobility. This outcome suggests that participation in even minimal physical activity may be enough to bring about small improvements in mobility; other intervention studies with older adults have reported similar findings (18,19).

This study had several limitations. Without a control group, outcomes must be interpreted with caution; because all participants were volunteers and not randomly selected, outcomes cannot be generalized to all older adults. Loss of data from 5 programs was greater than desired. Data from these programs were either not submitted or were lost. Future research should address the length and type of training needed to fully prepare lay leaders to conduct tests and gather and report outcome data appropriately.

Before this study, AMOB/VLL programs had been implemented primarily among white populations (12,13,17). Although 28% of the population in South Carolina is African American (20), we had double that percentage in AMOB/VLL programs. Compared with AMOB/VLL participants in other studies, our participants had less income and education. Our data suggest the potential value of evidence-based programs for a substantial proportion of older adults, African Americans, and those with less income and education. Our project provides data on successful implementation of the AMOB/VLL program among older African Americans and suggests that disadvantaged older adults of different races can benefit from such programs.

## Figures and Tables

**Table:** 

**1. The activity supported the learning objectives.**				
**Strongly Disagree**				**Strongly Agree**
1	2	3	4	5
**2. The material was organized clearly for learning to occur.**				
**Strongly Disagree**				**Strongly Agree**
1	2	3	4	5
**3. The content learned from this activity will impact my practice.**				
**Strongly Disagree**				**Strongly Agree**
1	2	3	4	5
**4. The activity was presented objectively and free of commercial bias.**				
**Strongly Disagree**				**Strongly Agree**
1	2	3	4	5

## Post-Test Information

To obtain credit, you should first read the journal article. After reading the article, you should be able to answer the following, related, multiple-choice questions. To complete the questions (with a minimum 70% passing score) and earn continuing medical education (CME) credit, please go to http://www.medscape.org/journal/pcd. Credit cannot be obtained for tests completed on paper, although you may use the worksheet below to keep a record of your answers. You must be a registered user on Medscape.org. If you are not registered on Medscape.org, please click on the "Register" link on the right hand side of the website to register. Only one answer is correct for each question. Once you successfully answer all post-test questions you will be able to view and/or print your certificate. For questions regarding the content of this activity, contact the accredited provider, CME@medscape.net. For technical assistance, contact CME@webmd.net. American Medical Association's Physician's Recognition Award (AMA PRA) credits are accepted in the US as evidence of participation in CME activities. For further information on this award, please refer to http://www.ama-assn.org/ama/pub/category/2922.html. The AMA has determined that physicians not licensed in the US who participate in this CME activity are eligible for **AMA PRA Category 1 Credits™**. Through agreements that the AMA has made with agencies in some countries, AMA PRA credit may be acceptable as evidence of participation in CME activities. If you are not licensed in the US, please complete the questions online, print the AMA PRA CME credit certificate and present it to your national medical association for review.

## Post-Test Questions

### Article Title: Evidence-Based Program Effectively Reduces Fear of Falling

**CME Questions**

1. You are a public health official asked to consult with the public health department of a South Carolina region with a predominantly African American underserved population regarding reducing fall risk among the elderly. Based on the study by Dr. Ullmann and colleagues, which of the following statements about implementing A Matter of Balance/Volunteer Lay Leader Model (AMOB/VLL) is **most likely** to appear in your report?
  - AMOB/VLL does not use a cognitive-behavioral approach
  - AMOB/VLL teaches that falls inevitably accompany aging
  - AMOB/VLL is designed for large groups of 50 or more participants
  - AMOB/VLL uses a 3-tiered organizational structure consisting of an active, engaged, and interactive state-level team, regional team, and local service providers
2. Based on the study by Dr. Ullmann and colleagues, which of the following statements about barriers and facilitators to implementation of AMOB/VLL is **most likely** to appear in your report?
  - Most of the teams had a high level of awareness about the availability and potential benefits of such programs
  - Lack of easy access to program sites was not a significant barrier to implementation
  - Facilitating factors included the state-level leadership in guiding the project and the history of the state, regional, and local groups working successfully together on other projects
  - The Area Agencies on Aging did not affect implementation
3. Based on the study by Dr. Ullmann and colleagues, which of the following statements about outcomes of implementing AMOB/VLL would **most likely** be correct?
  - Mobility was not significantly affected
  - Results of this study suggest that predominantly African American populations are unlikely to benefit from such programs
  - Results of this study suggest that populations containing many elderly adults of low income and low literacy are unlikely to benefit from such programs
  - The program was associated with greater confidence in managing falls and in carrying out activities of daily living

**Evaluation**
